# Taxonomic reassessment and color-pattern polymorphism of the Oriental fire-bellied newt *Hypselotriton orientalis* (Urodela, Salamandridae)

**DOI:** 10.3897/zookeys.1278.173183

**Published:** 2026-04-27

**Authors:** Zhihao Jiang, Jingjing Yang, Wei Zhao, Beibei He, Yue Pan, Song Huang, Jean Raffaëlli, Jinmin Chen

**Affiliations:** 1 The Anhui Provincial Key Laboratory of Biodiversity Conservation and Ecological Security in the Yangtze River Basin, College of Life Sciences, Anhui Normal University, Wuhu 241000, China The Anhui Provincial Key Laboratory of Biodiversity Conservation and Ecological Security in the Yangtze River Basin, College of Life Sciences, Anhui Normal University Wuhu China; 2 Penclen, Plumelec, 56420, France Unaffiliated Penclen France

**Keywords:** Dabie Mountains, morphology, new status, phylogeny, taxonomy, Yangtze River

## Abstract

The Oriental fire-bellied newt *Hypselotriton orientalis*, widely distributed in eastern China, has long faced taxonomic ambiguities. Although the nominate subspecies of *H. orientalis* and the other subspecies, *H. orientalis qianshan*, are known to exhibit geographic segregation, the taxonomic status and distribution pattern of these two groups remain unverified by integrated evidence and comprehensive sampling. In addition, the color-pattern polymorphism of this newt is poorly studied. Herein, based on extensive geographic sampling (128 samples, 19 localities), morphological examination, and phylogenetic analysis of mitochondrial NADH dehydrogenase subunit 2 (ND2) gene fragments, we elevate the Dabie Mountain region subspecies to distinct-species status as *H. qianshan***stat. nov**., and reassess its distribution range. Morphologically, this newly ranked taxon distinctly differs from *H. orientalis orientalis* by its smaller body length ratios (TOL/SVL and TAL/SVL), smaller fore-limb length ratio (FLL/SVL); longer internasal distance in males, longer interorbital distance in females; rust-red or rust-white patches on the dorsum; discontinuous dorsal ridge lines; the presence of orange-red patches on finger I; discontinuous or absent black stripes on the venter; and relatively inconspicuous vertebral ridge. Molecularly, the newly ranked taxon forms an independent clade with strong support (1.00/99) in the phylogenetic trees; the uncorrected group mean genetic distance between the newly ranked taxon and *H. orientalis orientalis* was 3.5%. Geographically, the newly ranked taxon and *H. orientalis orientalis* are separated by the Yangtze River, Luoxiao Mountains, Jiuling Mountains and Mufu Mountains. The number of species in the genus *Hypselotriton* now reaches 12. Meanwhile, we further report on the intraspecific color-pattern polymorphism of *H. orientalis* sensu lato. The potential species richness and color-pattern polymorphism in salamanders deserves further attention.

## Introduction

The fire-bellied newt genus *Hypselotriton* Wolterstorff, 1934, belongs to the family Salamandridae and is restricted to southern China (AmphibiaChina 2025). For the past forty years, the distinction between the genera *Cynops* Tschudi, 1838, and *Hypselotriton* has been the subject of an ongoing controversy (Zhao and Hu 1984; Zhao et al. 1988; Chan et al. 2001; Weisrock et al. 2006; Zhang et al. 2008; Dubois and Raffaëlli 2011; Raffaëlli 2013; Fei and Ye 2016; Hernandez 2018; Raffaëlli 2022). Recent phylogenetic studies suggest that the species of *Hypselotriton* from the Chinese mainland are monophyletic, whereas *Cynops* in its former configuration was paraphyletic with respect to *Pachytriton* and *Paramesotriton* (Yuan et al. 2022; Jiang et al. 2024; Wang et al. 2024). In this work, we adopt *Hypselotriton* at the generic level following the latest taxonomic arrangements (AmphibiaChina 2025; Frost 2025).

The genus *Hypselotriton* was erected by Wolterstorff (1934) for the species *H. wolterstorffi* (Boulenger, 1905). Within this genus, Zhao and Hu (1984) and Zhao et al. (1988) recognized two species-groups, and Dubois and Raffaëlli (2009, 2011) classified them into two subgenera. The nominotypical subgenus is *Hypselotriton*, which includes *Hypselotriton* (*H.*) chenggongensis (Kou & Xing, 1983), H. (H.) cyanurus (Liu, Hu & Yang, 1962), and H. (H.) wolterstorffi (Boulenger, 1905). The second subgenus is *Cynotriton*, which includes *Hypselotriton* (*C.*) fudingensis (Wu, Wang, Jiang & Hanken, 2010), H. (C.) orientalis (David, 1873), and H. (C.) orphicus (Risch, 1983). Yuan et al. (2013) described *H. glaucus* (Yuan, Jiang, Ding, Zhang & Che, 2013) from Guangdong as a new species based on morphological and molecular characters. Rao (2022) described *H. puerensis* (Rao, 2022) from Yunnan as a new species based on morphological characters. Lyu et al. (2023) described *H. jiaoren* (Lyu, Qi & Wang, 2023) and *H. maguae* (Lyu, Qi & Wang, 2023) as new species based on morphological and molecular characters; meanwhile, they elevated the subspecies *H. cyanurus yunnanensis* (Yang, 1983) to species rank and synonymized *H. puerensis* with *H. yunnanensis*. Jiang et al. (2024) described *H. huanggangensis* Jiang, Huang, Fan, Cheng, Raffaëlli & Chen, 2024 as a new species based on morphological and molecular characters. Wang et al. (2024) erected a new subgenus *Hakkatriton*, which included *Hypselotriton* (*H.*) orphicus and H. (H.) jiaoren, and described *H. oolong* Wang, Zeng, Wei & Lyu, 2024 as a new species based on morphological and molecular characters; meanwhile, they synonymized *H. glaucus* with *H. orphicus*. Currently, the following 11 species have been recorded: *Hypselotriton wolterstorffi*, *H. orientalis*, *H. orphicus*, *H. chenggongensis*, *H. cyanurus*, *H. fudingensis*, *H. huanggangensis*, *H. jiaoren*, *H. maguae*, *H. oolong* and *H. yunnanensis*.

The Oriental fire-bellied newt *Hypselotriton orientalis* (David, 1873) sensu lato is widely distributed in China, including Anhui, Jiangsu, Zhejiang, Fujian, Jiangxi, Hubei, Henan, and Hunan provinces (Fei and Ye 2016). Fei et al. (2012) described a new subspecies, *H. orientalis qianshan*, which is distributed in Anhui, Henan, and Hubei provinces, based on its smaller body size, smoother skin, weaker vertebral ridge, and thinner ventral fin fold compared to the nominate subspecies. At the molecular level, few phylogenetic studies have been conducted on *H. orientalis* sensu lato due to limited samples, and the phylogenetic relationships within the species remain unresolved (Wu et al. 2010a; Yuan et al. 2022). In this work, we elevate *Hypselotriton orientalis qianshan* to the rank of a distinct species based on molecular and morphological data.

Color-pattern polymorphism among many species has played an important role in evolution and ecology, and sometimes this polymorphism is taxon-specific (Giery et al. 2020; Smith et al. 2024). The genus *Hypselotriton* has several polymorphic species, but only a few have been studied (Fei et al. 2006; AmphibiaChina 2025). For decades, the description of *H. orientalis* sensu lato has been incomplete, often causing confusion in taxonomic morphological comparisons. In this work, we described the different color patterns of *H. orientalis* sensu lato.

## Materials and methods

### Sampling

**Figure 1.**
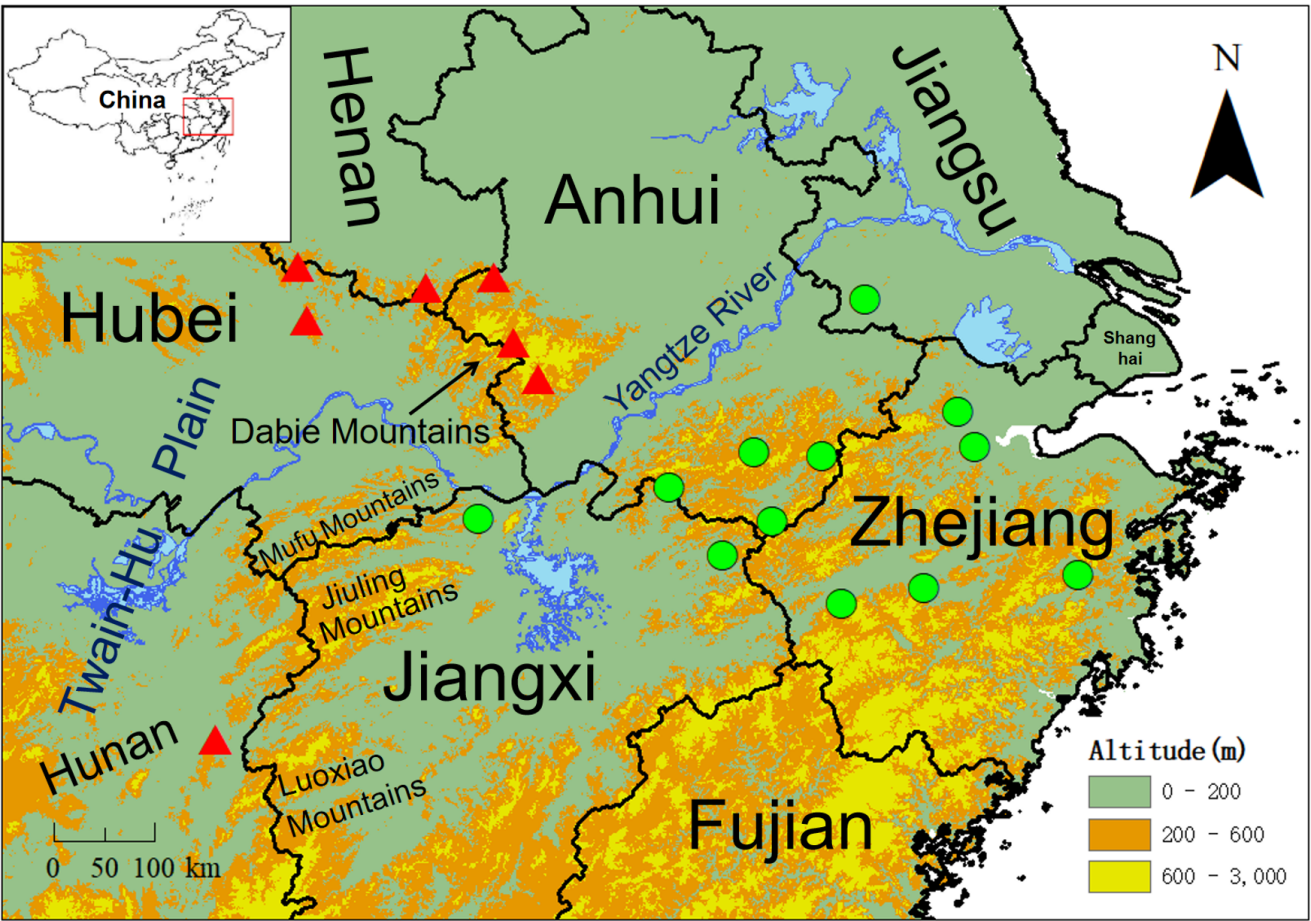
Sample collection of *Hypselotriton qianshan* stat. nov. (red triangles) and *H. orientalis orientalis* (green circles) in East-Central China. They are separated by the Yangtze River, Luoxiao Mountains, Jiuling Mountains and Mufu Mountains.

128 specimens of *Hypselotriton orientalis* sensu lato were collected or examined from 2021 to 2024 (Fig. 1). The toe tips of 18 specimens (the first toe of each specimen) were cut off and immediately preserved in 75% ethanol. These samples were then used for DNA analysis. After identifying that there is a specific phylogenetic relationship within *H. orientalis* sensu lato, 21 male and 26 female specimens were humanely euthanized by the injection of 0.7% tricaine methanesulfonate (MS222) solution (Yang et al. 2023), and fresh liver tissue was extracted and immediately preserved in 95% ethanol. The specimens were fixed in 10% formalin for 1 day, subsequently preserved in 75% ethanol and deposited in the Anhui Normal University Museum (voucher numbers: ANU240012-ANU2024058). Other living specimens are currently being kept in the laboratory of Anhui Normal University. Collections of all animals used in the present study comply with the Wildlife Protection Act of China, following the guidelines and regulations approved by the internal review board of Anhui Normal University (approval no. AHNU-ET2025004), and with the permissions of local government authorities.

### Morphological examinations

External measurements were made on 47 specimens of *Hypselotriton orientalis* sensu lato with digital calipers to the nearest 0.1 mm. Only adult specimens were measured (Lyu et al. 2023). These 14 measurements are as follows: total length (TOL) from tip of snout to tip of tail; snout–vent length (SVL) from tip of snout to posterior edge of vent; tail length (TAL) from posterior edge of vent to tip of tail; maximum tail depth (TAD); head length (HL) from tip of snout to the posterior edge of the parotoid gland; maximum head width (HW); snout length (SL) from tip of snout to anterior corner of eye; eye diameter (ED) from the anterior corner to the posterior corner of the eye; interorbital distance (IOD) between the anterior corner of each eye; eye–nostril length (EN) from the anterior corner of the eye to the nostril; internasal distance (IND) between the external nares; axilla–groin length (AG) between the axilla and the groin along the body; fore-limb length (FLL) from elbow to tip of finger III; and hind-limb length (HLL) from knee to tip of toe III. The color-pattern morphs of *H. orientalis qianshan* and *H. orientalis orientalis* were described separately by four relatively stable and distinctive characters of color pattern: ground color (black, dark brown or dark green), dorsal luster (conspicuous or weak), brownish-red middorsal stripes (continuous, interrupted, or absent), and dorsal spots/patches (black, rust-red/white, or absent).

Statistical analyses on the morphometric measurements were performed in IBM SPSS Statistics 27.0. Males and females were analyzed separately, due to obvious sexual size dimorphism (Fei et al. 1990; Fei and Ye 2016). Considering differences in growth rate and age among individuals, all morphometric data (except SVL) were divided by SVL to reduce the effect of differing growth stages among individuals. All measurements were normalized to reduce the variance (most P values > 0.05 in the Levene’s test). Univariate Analysis of Covariance (ANCOVA) with SVL as the covariate was used to test for differences between individuals of *Hypselotriton orientalis qianshan* and *H. orientalis orientalis* (Lai and Lue 2008).

### Molecular phylogeny

Total genomic DNA was extracted from 18 ethanol-preserved liver tissue samples, using the Qiagen DNEasy blood and tissue extraction kit (Qiagen Inc., Valencia, CA, USA). A 1026-bp fragment was amplified using primers KIZL4437 (Yuan et al. 2011) and 5081R (Wu et al. 2010b). Two internal primers, ND2–38R (5'–TATTCAYCCTAARTGTGCR–3') and 4416F (Wu et al. 2010b), were applied for sequencing (Yuan et al. 2013). Standard polymerase chain reactions (PCR) were performed in a final volume of 15 μl with the following procedures: initial denaturation at 94 °C for 5 min, 35 amplification cycles at 94 °C for 1 min, annealing for 1 min at 52 °C, extension for 1 min at 72 °C. The final extension at 72 °C was conducted for 10 min. The successfully amplified products were purified using ExoSAP-IT purification kit according to the manufacturer’s instructions (Yuan et al. 2013). Purified PCR products were directly sequenced in both directions using a BigDye Terminator Cycle Sequencing Kit (v. 2.0, Applied Biosystems, Foster City, California, USA) and an ABI PRISM 3730 automated DNA sequencer (Yuan et al. 2013). The phylogenetic relationships within *Hypselotriton* were derived from an analysis of the mtDNA fragment that codes for subunit 2 of NADH dehydrogenase (ND2) and its flanking tRNAs. *Pachytriton archospotus* Shen, Shen & Mo, 2008 was chosen as the outgroup species. In addition, 25 sequences were also included based on previous studies and obtained from GenBank (Wu et al. 2010a; Yuan et al. 2016; Yuan et al. 2022; Lyu et al. 2023; Jiang et al. 2024; Wang et al. 2024). Detailed information is provided in Table 1. DNA sequences were aligned using MEGA v. 11 (Kumar et al. 2018) with ClustalW under default parameters and manually checked.

**Table 1.** Localities, voucher information, and GenBank accession numbers for all *Hypselotriton* samples of ND2 used in this study.

ID	Species name	Localities	Voucher	ND2	Source
1	*H. qianshan* stat. nov.	China: Anhui: Jinzhai	HSA24170	PV090941	this study
2	*H. qianshan* stat. nov.	China: Hunan: Zhuzhou	HSA24116	PV090935	this study
3	*H. qianshan* stat. nov.	China: Hubei: Xiaogan	HSA24164	PV090937	this study
4	*H. qianshan* stat. nov.	China: Hunan: Zhuzhou	HSA24115	PV090934	this study
5	*H. qianshan* stat. nov.	China: Hubei: Xiaogan	HSA24165	PV090938	this study
6	*H. qianshan* stat. nov.	China: Henan: Shangqiu	HSA24162	PV090936	this study
7	*H. qianshan* stat. nov.	China: Anhui: Yuexi	HSA24166	PV090939	this study
8	*H. qianshan* stat. nov.	China: Anhui: Yuexi	HSA24167	PV090940	this study
9	*H. qianshan* stat. nov.	China: Henan: Jigongshan	KIZ 013021	ON793736	Yuan et al. 2022
10	*H. orientalis*	China: Anhui: Dafu	CJM699	PV090923	this study
11	*H. orientalis*	China: Anhui: Dafu	KIZ 021962	ON793737	Yuan et al. 2022
12	*H. orientalis*	China: Zhejiang: Hangzhou	HSA24117	PV090928	this study
13	*H. orientalis*	China: Jiangsu: NanJing	HSA24118	PV090929	this study
14	*H. orientalis*	China: Jiangsu: NanJing	HSA24119	PV090930	this study
15	*H. orientalis*	China: Anhui: Jixi	CJM1735	PV090924	this study
16	*H. orientalis*	China: Zhejiang: Quzhou	CIB97919	GU301790	Wu et al. 2010a
17	*H. orientalis*	China: Zhejiang: Jinhua	KIZ06358	ON793718	Yuan et al. 2022
18	*H. orientalis*	China: Zhejiang: Tiantai	KIZ 012941	ON793732	Yuan et al. 2022
19	*H. orientalis*	China: Zhejiang: Deqing	HSA24113	PV090927	this study
20	*H. orientalis*	China: Zhejiang: Jinhua	HSA24111	PV090926	this study
21	*H. orientalis*	China: Jiangxi: Jiujiang	KIZ 020539	ON793738	Yuan et al. 2022
22	*H. orientalis*	China: Jiangxi: Jiujiang	KIZ 020536	ON793739	Yuan et al. 2022
23	*H. orientalis*	China: Anhui: Jiulongfeng	CJM1859	PV090925	this study
24	*H. orientalis*	China: Anhui: Shanli	HSA24169	PV090932	this study
25	*H. orientalis*	China: Jiangxi: Wuyuan	YPX25002	ON793740	Yuan et al. 2022
26	*H. orientalis*	China: Anhui: Shanli	HSA24171	PV090933	this study
27	*H. huanggangensis*	China: Jiangxi: Yanshan	HSA23075	PP590780	Jiang et al. 2024
28	*H. huanggangensis*	China: Jiangxi: Yanshan	HSA23076	PP590788	Jiang et al. 2024
29	*H. orphicus*	China: Guangdong: Jiexi	GEP a008	PP986999	Wang et al. 2024
30	*H. orphicus*	China: Guangdong: Jiexi	GEP a009	PP987000	Wang et al. 2024
31	*H. fudingensis*	China: Fujian: Ningde	CIB 97874	GU301785	Wu et al. 2010a
32	*H. fudingensis*	China: Fujian: Ningde	SYS a008487	OQ116688	Lyu et al. 2023
33	*H. jiaoren*	China: Guangdong: Yingde	SYS a008786	OQ116679	Lyu et al. 2023
34	*H. jiaoren*	China: Guangdong: Yingde	SYS a008787	OQ116680	Lyu et al. 2023
35	*H. maguae*	China: Jiangxi: Fuzhou	CIB 118535	OQ116685	Lyu et al. 2023
36	*H. maguae*	China: Jiangxi: Fuzhou	SYS a007032	OQ116686	Lyu et al. 2023
37	*H. cyanurus*	China: Guizhou: Liupanshui	CIB 95897	GU301784	Wu et al. 2010a
38	*H. cyanurus*	China: Guizhou: Liupanshui	KIZ 02331	ON793754	Yuan et al. 2022
39	*H. yunnanensis*	China: Yunnan: Chuxiong	KIZ 021922	ON793749	Yuan et al. 2022
40	*H. yunnanensis*	China: Yunnan: Kunming	KIZ 022160	ON793752	Yuan et al. 2022
41	*H. oolong*	China: Guangdong: Chaozhou	CIB 121429	PP987004	Wang et al. 2024
42	*H. oolong*	China: Guangdong: Chaozhou	CIB 121430	PP987005	Wang et al. 2024
43	*Pachytriton archospotus*	China: Hunan: Guidong	KIZ 04563	KU375007	Yuan et al. 2016

The matrilineal genealogy was reconstructed using Bayesian-inference (BI) and maximum-likelihood (ML) methods based on the ND2 gene. PartitionFinder2 was used to test the best-fitting partitioning scheme and nucleotide substitution model. The data were analyzed using BI in MrBayes v. 3.2.4 (Ronquist et al. 2012), and ML in RaxmlGUI v. 1.3 (Silvestro and Michalak 2012). Two independent runs were conducted in the BI analysis, each run for 10 million generations and sampled every 1000 generations, with the first 25% of the samples discarded as burn-in. The analyses used the proportion of invariable sites estimated from the data and 1000 bootstrap pseudoreplicates under the GTR+gamma model (Chen et al. 2021). Bayesian posterior probabilities (BPP) and ML bootstrap support (BS) were labeled around the tree nodes. Mean genetic distances between *Hypselotriton orientalis qianshan* and *H. o. orientalis* species were calculated in MEGA v. 11 using the uncorrected genetic distance model.

## Results

Morphologically, this newly ranked taxon distinctly differs from *Hypselotriton orientalis orientalis* by its rust-red or rust-white patches on the dorsum, discontinuous dorsal ridge lines, orange-red patches on finger I, black stripes on the venter of the neck, absent or discontinuous, and a relatively inconspicuous vertebral ridge (Figs 3, 4, 5, 6). Statistical analyses on the morphometric measurements (14 measurements) were performed using the specimens of *H. qianshan* stat. nov. and *H. orientalis orientalis* (Tables 2, 3). The results of linear measurements and Univariate Analysis of Covariance (ANCOVA) with SVL as the covariate show that the newly ranked taxon has a smaller body length ratio (TOL/SVL and TAL/SVL), smaller fore-limb length ratio (FLL/SVL), longer internasal distance in males, and longer interorbital distance in females. Descriptions of color-pattern polymorphism show that *H. orientalis* sensu lato has significant polymorphisms at the intraspecific level (details in Taxonomic account below).

**Table 2.** Linear measurements (in mm) of *Hypselotriton orientalis* sensu lato: all morphometric data (except SVL) were divided by SVL to reduce the effect of different growth stages in different individuals. Abbreviations are provided in the text. Data in “()” means Mean±SE. Detailed measurements are listed in Suppl. material 1.

	*H. qianshan* stat. nov.		*H. orientalis*	
	Male (*N* = 10)	Female (*N* = 7)	Male (*N* = 16)	Female (*N* = 14)
TOL	1.52–1.72(1.65 ± 0.02)	1.62–1.83(1.71 ± 0.03)	1.66–1.76(1.71 ± 0.01)	1.73–1.84(1.77 ± 0.01)
TAL	0.52–0.72(0.65 ± 0.02)	0.62–0.83(0.71 ± 0.03)	0.66–0.76(0.71 ± 0.01)	0.73–0.84(0.77 ± 0.01)
TAD	0.08–0.15(0.12 ± 0.01)	0.08–0.12(0.10 ± 0.01)	0.08–0.15(0.12 ± 0.00)	0.09–0.14(0.11 ± 0.00)
HL	0.24–0.30(0.28 ± 0.01)	0.25–0.30(0.27 ± 0.01)	0.25–0.31(0.28 ± 0.00)	0.24–0.31(0.27 ± 0.00)
HW	0.17–0.22(0.20 ± 0.01)	0.18–0.25(0.20 ± 0.01)	0.18–0.21(0.19 ± 0.00)	0.17–0.21(0.19 ± 0.00)
SL	0.07–0.10(0.09 ± 0.00)	0.06–0.09(0.08 ± 0.00)	0.08–0.10(0.09 ± 0.00)	0.06–0.10(0.08 ± 0.00)
ED	0.05–0.08(0.06 ± 0.00)	0.05–0.07(0.06 ± 0.00)	0.06–0.12(0.07 ± 0.00)	0.05–0.07(0.06 ± 0.00)
IOD	0.11–0.13(0.12 ± 0.00)	0.10–0.12(0.11 ± 0.00)	0.06–0.12(0.11 ± 0.00)	0.10–0.11(0.10 ± 0.00)
EN	0.06–0.08(0.07 ± 0.00)	0.06–0.07(0.06 ± 0.00)	0.06–0.08(0.07 ± 0.00)	0.05–0.07(0.06 ± 0.00)
IND	0.05–0.06(0.05 ± 0.00)	0.04–0.05(0.04 ± 0.00)	0.04–0.06(0.05 ± 0.00)	0.04–0.06(0.05 ± 0.00)
AG	0.42–0.51(0.48 ± 0.01)	0.43–0.54(0.49 ± 0.01)	0.41–0.51(0.46 ± 0.01)	0.46–0.61(0.50 ± 0.01)
FLL	0.30–0.36(0.33 ± 0.01)	0.26–0.34(0.31 ± 0.01)	0.30–0.37(0.34 ± 0.01)	0.28–0.35(0.32 ± 0.01)
HLL	0.32–0.37(0.34 ± 0.01)	0.28–0.34(0.31 ± 0.01)	0.29–0.38(0.34 ± 0.01)	0.29–0.36(0.33 ± 0.01)

**Table 3.** Test for differences between *Hypselotriton qianshan* stat. nov. and *H. orientalis orientalis* using ANCOVA (SVL as covariate).

Variable	Males	Females
Total length (TOL)		
*F*	6.83	7.95
*P*	0.016	0.011
Tail length (TAL)		
*F*	6.83	7.95
*P*	0.016	0.011
Maximum tail depth (TAD)		
*F*	2.65	0.32
*P*	0.117	0.581
Head length (HL)		
*F*	2.83	0.03
*P*	0.106	0.867
Maximum head width (HW)		
*F*	0.62	1.98
*P*	0.439	0.176
Snout length (SL)		
*F*	0.05	0.05
*P*	0.825	0.835
Eye diameter (ED)		
*F*	2.39	1.03
*P*	0.136	0.323
Interorbital distance (IOD)		
*F*	2.28	9.31
*P*	0.145	0.007
Eye–nostril length (EN)		
*F*	0.06	0.45
*P*	0.809	0.510
Internasal distance (IND)		
*F*	4.95	1.00
*P*	0.036	0.331
Axilla–groin length (AG)		
*F*	2.01	0.26
*P*	0.170	0.616
Fore-limb length (FLL)		
*F*	6.14	0.93
*P*	0.021	0.349
Hind-limb length (HLL)		
*F*	0.39	4.36
*P*	0.539	0.051

**Figure 2.**
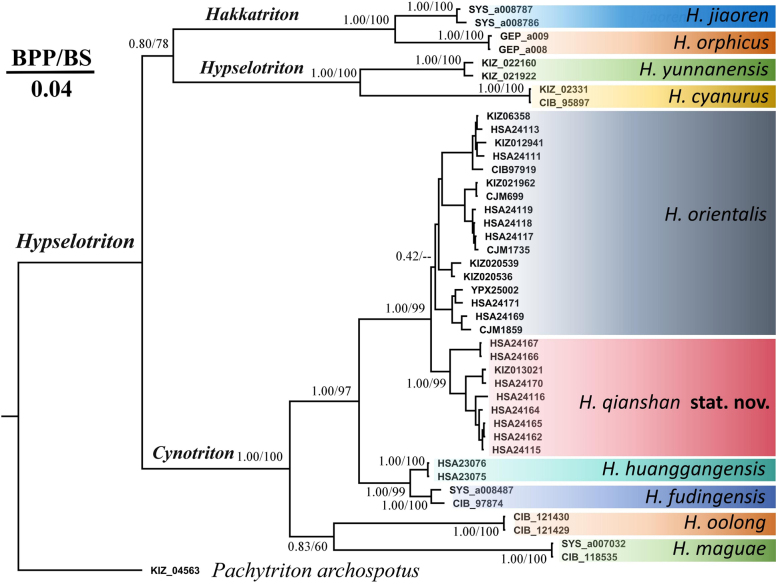
Bayesian-inference tree and maximum-likelihood phylogenies based on the mitochondrial ND2 gene. Bayesian posterior probabilities and the bootstrap supports (BPP/BS) are shown near the nodes. Numbers at the ends of the lineages correspond to the vouchers in Table 1.

Molecularly, BI and ML analyses resulted in similar topologies (Fig. 2). Within the subgenus *Cynotriton*, two major clades were revealed in samples of *Hypselotriton orientalis* sensu lato. The newly ranked taxon forms an independent clade with strong support (1.00/99). BPP and BS of the other clade (*H. orientalis orientalis*) are weak. The uncorrected group mean genetic distance between *H. qianshan* stat. nov. and *H. orientalis orientalis* was 3.5%. As shown in the tree, the Hunan population and the Dabie Mountain populations form a monophyletic group.

Accordingly, based on the results of the morphological examination presented below and the phylogenetic analysis, the specimens from the Dabie Mountains and Hunan Province are redescribed and elevated to species rank.

### Taxonomic account

#### Hypselotriton (Cynotriton) qianshan

Taxon classificationAnimaliaCaudataSalamandridae

(Fei, Ye & Jiang, 2012) stat. nov.

487C7E4B-36E0-5974-9577-E9EF6BA59CFC

##### Type.

***Holotype*** [not seen]. (ClB)74II0752, adult male from Qianshan City (30°37'N, 116°34'E; altitude 600 m), Anhui Province, China (Fei et al. 2012).

##### Material examined.

China – **Anhui Prov**. • 2 ♀♀; Jinzhai; 23 Oct. 2023; H. Xue leg.; AHNU • 1 ♀, 5 ♂♂; Yuexi; 15 Apr. 2024; Z.H. Jiang leg.; AHNU • 1 ♀; Huoshan; 17 Jul. 2021; Z.H. Jiang leg.; AHNU – **Henan Prov**. • 10 ♀♀, 2 ♂♂; Shangqiu; 27 Aug. 2024; Z.H. Jiang leg.; AHNU • 1 ♀; Jigongshan; 28 Aug. 2024; Z.H. Jiang leg.; AHNU – **Hubei Prov**. • 9 ♀♀, 10 ♂♂; Xiaogan; 13 Sep. 2024; Z.H. Jiang leg.; AHNU – **Hunan Prov**. • 5 ♂♂; Zhuzhou; 19 Sep. 2024; Z.H. Jiang leg.; AHNU.

##### Etymology.

The specific name qianshan, a noun in apposition, is the name of the type locality, Qianshan City, in Anhui Province (Fei et al. 2012). Proposed English name Qianshan fire-bellied newt. Proposed Chinese name 潜山蝾螈 (qián shān róng yuán).

##### Diagnosis.

(1) small body size, TOL 63.0–81.2 mm in adult males, TOL 65.1–99.9 mm in adult females; (2) head flat, without ridges on the sides; (3) parotoid gland inconspicuous; (4) postocular orange spot absent; (5) gular fold present; (6) vertebral ridge weak; (7) fingers and toes overlapping when fore-limb and hind-limb adpressed towards each other along body; (8) orange-red patches on finger I; and (9) ground color of venter and chin bright orange with irregular black patches, bright orange blotches on base of ventral limbs and anterior half of cloaca.

##### Color-pattern polymorphism.

**Figure 3.**
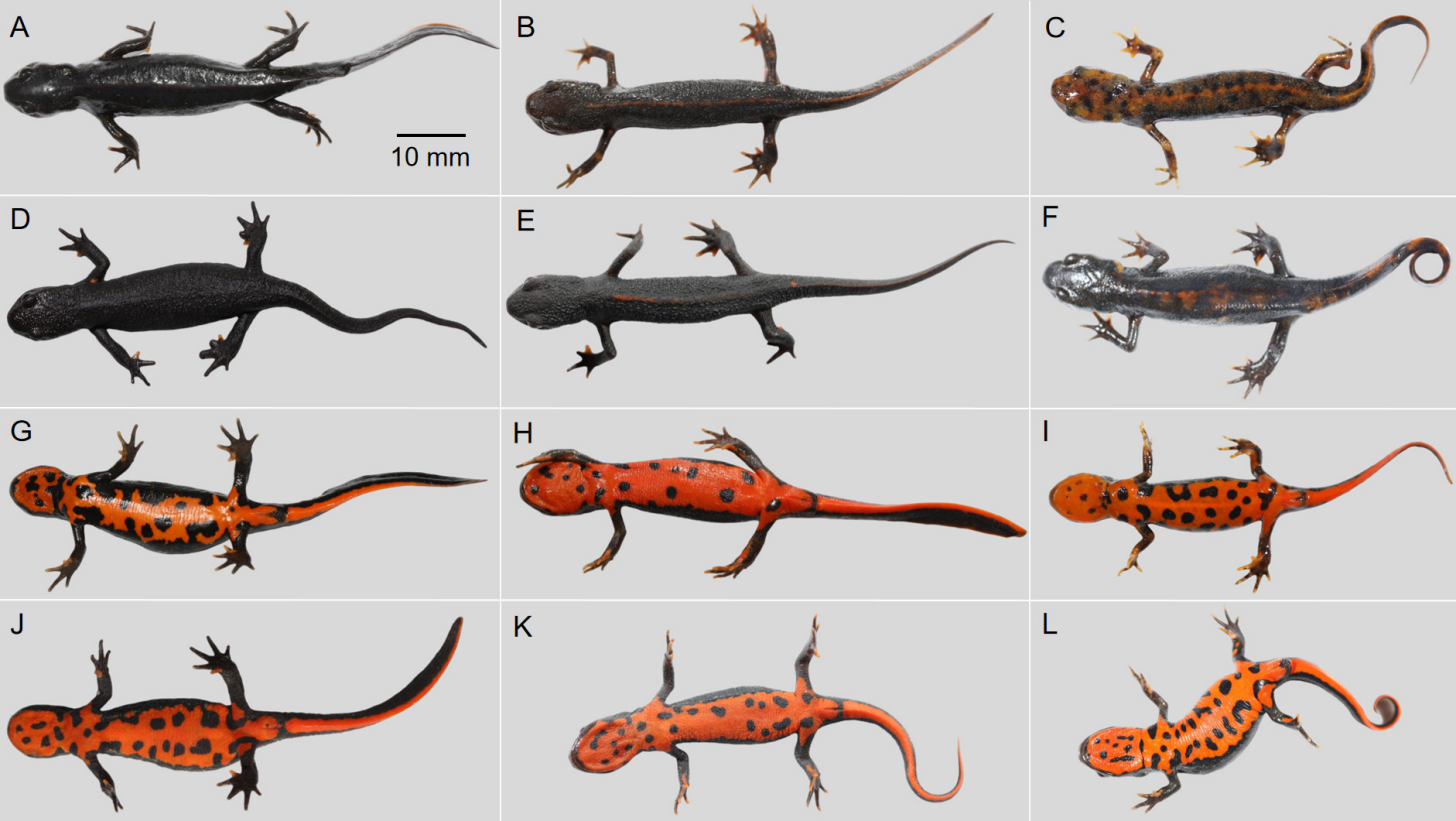
Color-pattern polymorphism of *Hypselotriton qianshan* stat. nov. **A–F**. Dorsal view; **G–L**. Ventral view. **A, G**.Yuexi, Anhui; **B, C, E, F, H, I, K, L**. Xiaogan, Hubei; **D, J**. Huoshan, Anhui. Scale bar in A applies to all.

The specimens of *Hypselotriton qianshan* stat. nov. were divided into six groups based on their dorsal color-pattern polymorphism (Fig. 3): (Fig. 3A) ground color black, dorsal luster conspicuous, brownish-red middorsal stripe absent, dorsal spots/patches absent; (Fig. 3B) ground color dark brown, dorsal luster weak, brownish-red middorsal stripe continuous, dorsal spots/patches absent; (Fig. 3C) ground color dark green, dorsal luster weak, brownish-red middorsal stripe continuous, dorsal spots black; (Fig. 3D) ground color black, dorsal luster weak, brownish-red middorsal stripe absent, dorsal spots/patches absent; (Fig. 3E) ground color dark brown, dorsal luster weak, brownish-red middorsal stripe interrupted, dorsal spots/patches absent; and (Fig. 3F) ground color black, dorsal luster conspicuous, brownish-red middorsal stripe absent, dorsal patches rust-red/white.

##### Comparisons.

*Hypselotriton qianshan* stat. nov. is phylogenetically close to *H. orientalis orientalis*, which is distributed in Anhui, Jiangsu, Zhejiang, Fujian and Jiangxi. However, *Hypselotriton qianshan* stat. nov. differs from *H. orientalis orientalis* by its orange-red patches on finger I (vs orange-red patches absent or inconspicuous) and by black stripes on the ventral neck being absent or discontinuous in most individuals (vs present and continuous in most individuals).

In addition, *Hypselotriton qianshan* stat. nov. further differs from other species of *Hypselotriton*, as follows:

*Hypselotriton qianshan* stat. nov. differs from *H. fudingensis* by its weak vertebral ridge (vs vertebral ridge conspicuous) and ventral black patches (vs venter bright orange without dark blotches).

*Hypselotriton qianshan* stat. nov. differs from *H. huanggangensis* by its orange-red patches on finger I (vs orange-red patches absent) and brownish-red middorsal stripe absent or relatively conspicuous (vs relatively inconspicuous).

*Hypselotriton qianshan* stat. nov. differs from *H. maguae* by having its fingers and toes overlapping when forelimbs and hindlimbs are adpressed (vs forelimbs and hindlimbs not meeting when adpressed towards each other along body) and gular fold present (vs gular fold absent).

*Hypselotriton qianshan* stat. nov. differs from *H. oolong* by its weak vertebral ridge (vs vertebral ridge conspicuous).

*Hypselotriton qianshan* stat. nov. differs from *H. orphicus* by bright orange patches on ventral forearms being absent (vs present).

*Hypselotriton qianshan* stat. nov. differs from *H. jiaoren* by gular fold present (vs gular fold absent).

*Hypselotriton qianshan* stat. nov. differs from *H. wolterstorffi*, *H. cyanurus*, *H. chenggongensis* and *H. yunnanensis* by its absent postocular orange spot (vs present).

##### Distribution.

Eastern China, including western Anhui, eastern Hubei, southeastern Henan and Hunan provinces.

##### Note on habitat.

The newt *Hypselotriton orientalis* sensu lato lives in the mountains at an altitude of 30–1000 m. It is generally found in rice fields or puddles. Previous records have mentioned that *H. qianshan* stat. nov. also inhabits slow-flowing shallow mountain streams with aquatic vegetation (Fei et al. 2012; Fei and Ye 2016). Considering the ability of *H. orientalis* sensu lato to adapt to new habitats and the ecological vulnerability of rice fields and puddles, the populations of *H. qianshan* stat. nov. in mountain streams deserves further attention.

#### Hypselotriton (Cynotriton) orientalis

Taxon classificationAnimaliaCaudataSalamandridae

(David, 1873) stat. nov.

246C7D25-E9A5-5958-89E3-55F5A1EDC9A0

##### Material examined.

China – **Anhui Prov**. • 2 ♀♀, 3 ♂♂; Shanli; 2 May 2024; Z.H. Jiang leg.; AHNU • 5 ♀♀, 12 ♂♂; Jiulongfeng; 19 Jul. 2022; J.M. Cheng leg.; AHNU • 2 ♂♂; Xuancheng; 13 Nov. 2022; J.M. Cheng leg.; AHNU • 3 ♂♂; Dafu; 13 Nov. 2023; J.M. Cheng leg.; AHNU – **Jiangxi Prov**. • 1 ♀; Shangrao; 2 Sep. 2021; Z.H. Jiang leg.; AHNU • 4 ♀♀, 5 ♂♂; Jiujiang; 20 Aug. 2021; Z.H. Jiang leg.; AHNU – **Jiangsu Prov**. • 4 ♀♀, 5 ♂♂; NanJing; 14 Sep. 2024; T.R. Zhang leg.; AHNU – **Zhejiang Prov**. • 5 ♀♀, 7 ♂♂; Hangzhou; 8 Oct. 2024; Z.H. Jiang leg.; AHNU • 4 ♀♀, 3 ♂; Deqing; 14 Sep. 2024; Z.H. Jiang leg.; AHNU • 3 ♀♀; Quzhou; 19 Oct. 2024; Z.H. Jiang leg.; AHNU • 1 ♀, 6 ♂♂; Jinhua; 12 Nov. 2024; Z.H. Jiang leg.; AHNU • 7 ♂♂; Tiantai; 10 Aug. 2024; Z.H. Jiang leg.; AHNU.

##### Diagnosis.

(1) small body size, TOL 61.2–75.4 mm in adult males, TOL 61.6–88.9 mm in adult females; (2) head flat, without ridges on the sides; (3) parotoid gland inconspicuous; (4) postocular orange spot absent; (5) gular fold present; (6) vertebral ridge weak or slightly bulged; (7) fingers and toes overlapping when fore-limb and hind-limb adpressed towards each other along body; (8) orange-red patches on finger I absent or inconspicuous; and (9) ground color of venter and chin bright orange with irregular black patches, bright orange blotches on base of ventral limbs and anterior half of cloaca.

##### Color-pattern polymorphism.

**Figure 4.**
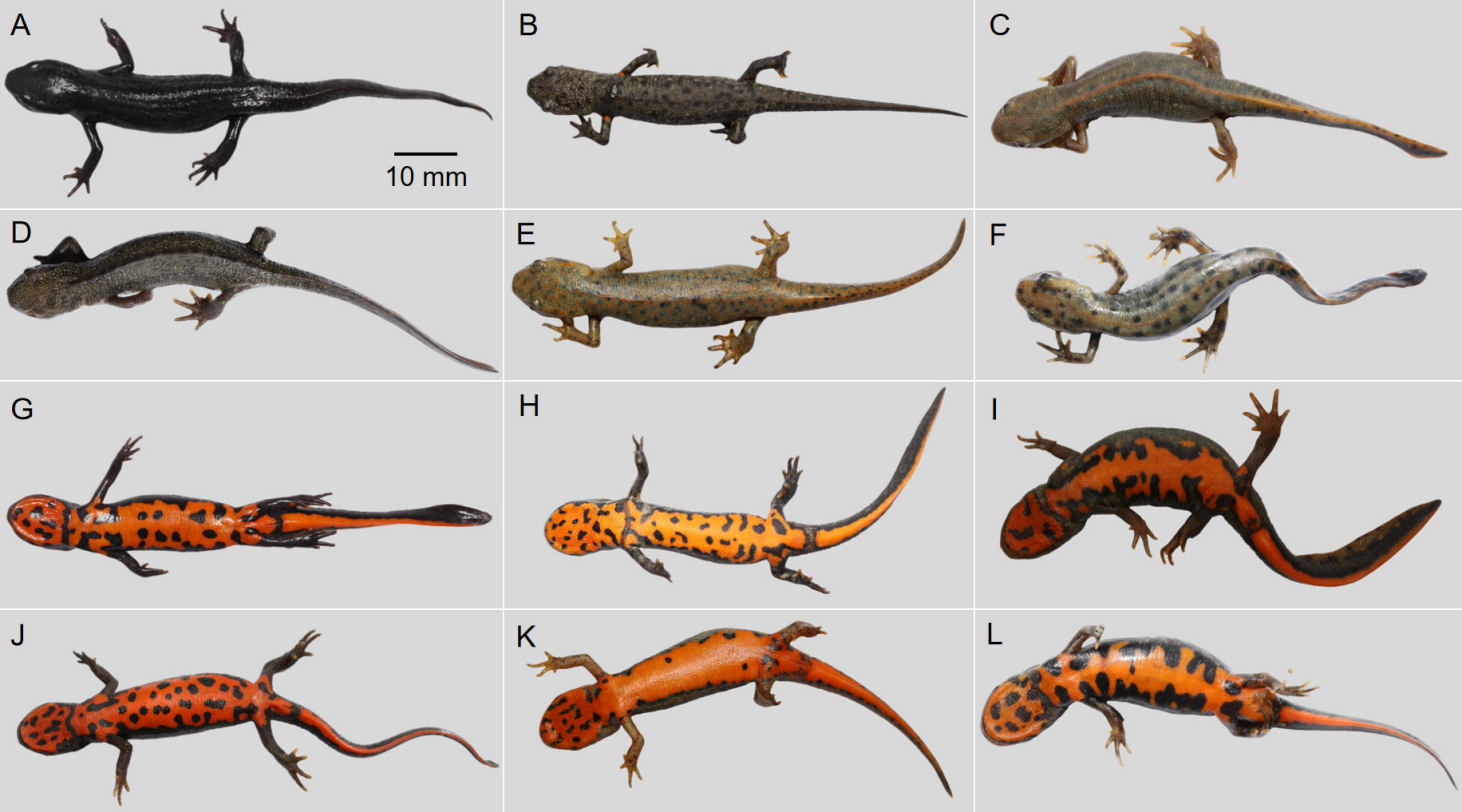
Color-pattern polymorphism of *Hypselotriton orientalis orientalis*. **A–F**. Dorsal view; **G–L**. Ventral view. **A, G, B, H, E, K**. Huangshan, Anhui; **C, I**. Quzhou, Zhejiang; **D, J**. Hangzhou, Zhejiang; **F, L**. Jinhua, Zhejiang. Scale bar in A applies to all.

The specimens of *Hypselotriton orientalis orientalis* were divided into six groups based on their dorsal color-pattern polymorphism (Fig. 4): ground color black, dorsal luster conspicuous, brownish-red middorsal stripe absent, dorsal spots/patches absent (Fig. 4A); ground color dark brown, dorsal luster weak, brownish-red middorsal stripe absent, dorsal spots black (Fig. 4B); ground color dark green, dorsal luster weak, brownish-red middorsal stripe continuous, dorsal spots black (Fig. 4C); ground color dark brown, dorsal luster weak, brownish-red middorsal stripe absent, dorsal spots/patches absent (Fig. 4D); ground color dark green, dorsal luster weak, brownish-red middorsal stripe absent, dorsal spots blue-green Fig. 4E); ground color dark green, dorsal luster conspicuous, brownish-red middorsal stripe continuous, dorsal spots black (Fig. 4F).

##### Comparisons.

*Hypselotriton orientalis orientalis* differs from *H. fudingensis* by orange-red patches on finger I being absent or inconspicuous (vs conspicuous) and ventral black patches (vs venter bright orange without dark blotches).

*Hypselotriton orientalis orientalis* differs from *H. huanggangensis* by black patches with clear boundaries on lateral trunk being absent or inconspicuous (vs conspicuous) and brownish-red middorsal stripe absent or conspicuous (vs inconspicuous).

*Hypselotriton orientalis orientalis* differs from *H. maguae* by having its fingers and toes overlapping when forelimbs and hindlimbs are adpressed (vs forelimbs and hindlimbs not meeting when adpressed towards each other along body) and gular fold present (vs gular fold absent).

**Figure 5.**
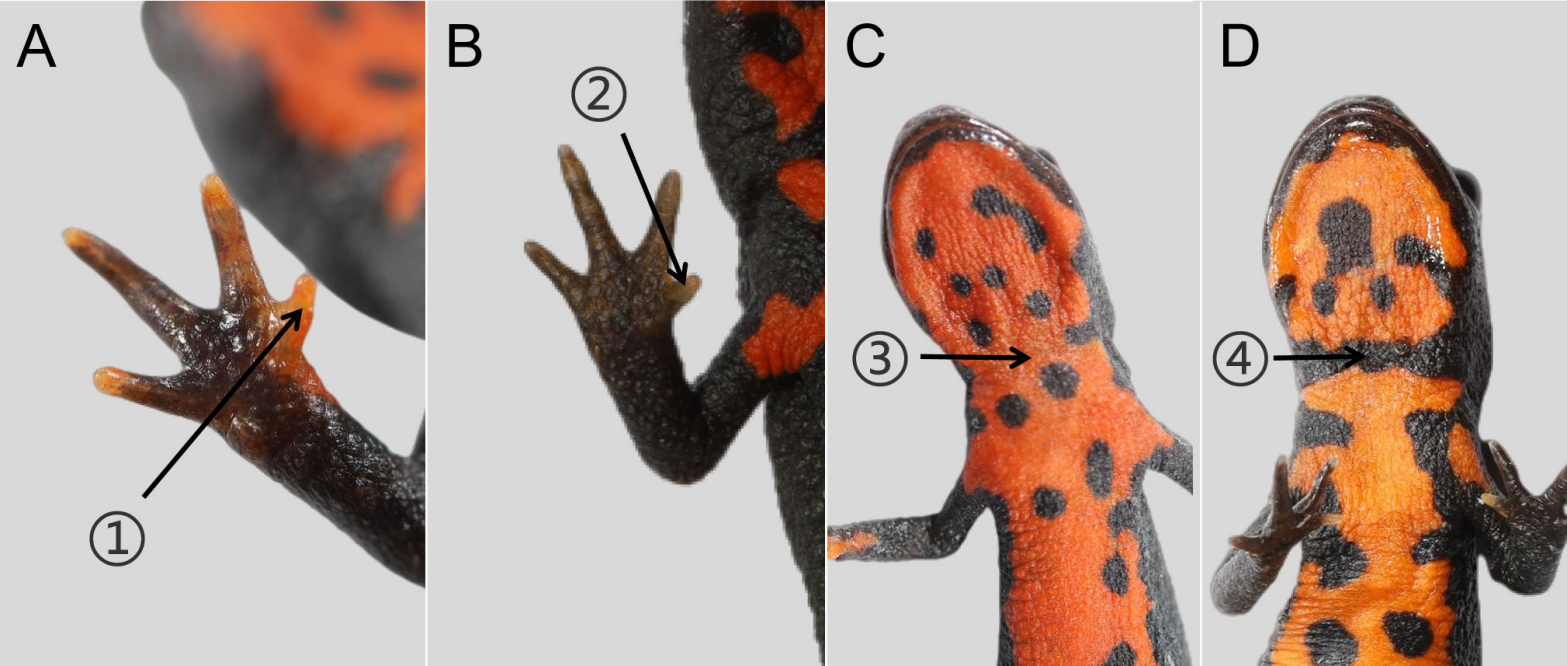
Comparison of *Hypselotriton qianshan* stat. nov. (**A, C**) and *H. orientalis orientalis* (**B, D**). Arrows indicate: 1 orange-red patches on finger I; 2 orange-red patches on finger I absent; 3 black stripes on the ventral neck absent; and 4 black stripe on the ventral neck continuous.

*Hypselotriton orientalis orientalis* differs from *H. oolong* by its orange-red patches on finger I absent or inconspicuous.

**Figure 6.**
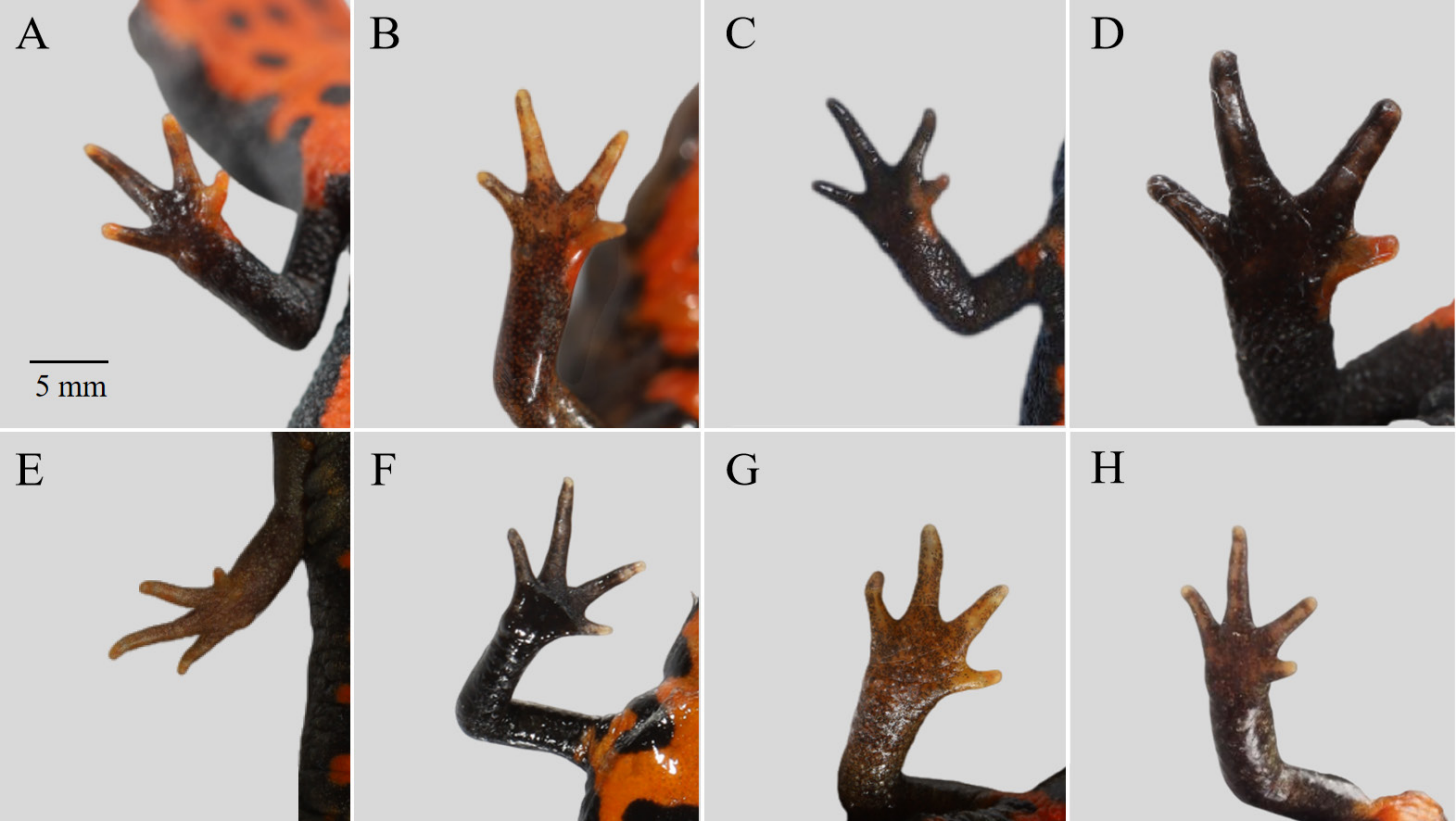
Fingers (ventral view) of *Hypselotriton qianshan* stat. nov. (**A–D**) and *H. orientalis orientalis* (**E–H**) from different areas. **A**. Xiaogan, Hubei; **B**. Shangqiu, Henan; **C**. Yuexi, Anhui; **D**. Jinzhai, Anhui; **E**. Quzhou, Zhejiang; **F**. Hangzhou, Zhejiang; **G**. NanJing, Jiangsu; **H**. Huangshan, Anhui. Scale bar in A applies to all.

*Hypselotriton orientalis orientalis* differs from *H. orphicus* by its bright orange patches on ventral forearms absent (vs present) and orange-red patches on finger I absent or inconspicuous (vs conspicuous).

*Hypselotriton orientalis orientalis* differs from *H. jiaoren* by its gular fold (vs gular fold absent).

*Hypselotriton orientalis orientalis* differs from *H. wolterstorffi*, *H. cyanurus*, *H. chenggongensis* and *H. yunnanensis* by its absent postocular orange spot (vs present).

##### Distribution.

Eastern China, including southern Anhui, southwestern Jiangsu, northern Fujian, northern Jiangxi and Zhejiang provinces.

## Discussion

The indefinite nature of many species boundaries has long been recognized (Burbrink et al. 2022). In this study, we elevate the subspecies *Hypselotriton orientalis qianshan* to a distinct-species status as *H. qianshan* stat. nov. based on morphological characters, phylogenetic evidence, and geographic separation, following the phylogenetic species concept and considering geographic separation an important factor in the process of speciation.

Previous biogeographic studies have shown that *Hypselotriton qianshan* stat. nov. and *H. orientalis orientalis* are predominantly isolated by the Yangtze River (Fei et al. 2012; Fei and Ye 2016). However, our phylogenetic results showed that the Hunan population and other populations north of the Yangtze River form a monophyletic group. *Hypselotriton orientalis* sensu lato was estimated to have diverged around the Pleistocene (Yuan et al. 2022). During that time, the Twain-Hu Plain was a flood plain composed of inter-river depressions (Li et al. 2001; Hu et al. 2010). *Hypselotriton qianshan* stat. nov. may have spread across the Twain-Hu Plain through numerous marshes, which is consistent with the phylogenetic results.

A combination of two or more characters (shorter trunk ratio, orange-red patches on finger I and black stripes on the venter of the neck, absent or discontinuous) can effectively distinguish *Hypselotriton qianshan* stat. nov. from *H. orientalis orientalis* (Fig. 5). Meanwhile, in the populations of *H. orientalis orientalis*, rough skin and conspicuous vertebral ridges are often found in northern Zhejiang and Jiangsu Province. In the populations of *H. qianshan* stat. nov., rust-red or rust-white patches on the dorsum and discontinuous dorsal ridge lines are often found in the western Dabie Mountains.

The Dabie Mountains are located in the transition zone between the Palearctic and Oriental realms, and have a high biological diversity. Several new species of amphibians have been discovered in the Dabie Mountains region in recent years, such as *Microhyla dabieshanensis* Zhang, Chen & Zhang, 2022, *Rana dabieshanensis* Wang, Qian, Zhang, Guo, Pan, Wu, Wang & Zhang, 2017, *Zhangixalus zhoukaiyae* (Pan, Zhang & Zhang, 2017) and *Tylototriton anhuiensis* Qian, Sun, Li, Guo, Pan, Kang, Wang, Jiang, Wu & Zhang, 2017 (Wang et al. 2017; Pan et al. 2017; Qian et al. 2017; Zhang et al. 2022). The newly ranked taxon *Hypselotriton qianshan* stat. nov. is also predominantly distributed in this region, where the potential species richness deserves further attention.

The potential drivers of color-pattern polymorphisms in *Hypselotriton orientalis* sensu lato remain unresolved. Notably, during captivity, some individuals developed slightly rougher skin after being transferred to a terrestrial environment, while others exhibited changes in dorsal coloration in response to different feeding conditions. These observations suggest that *H. orientalis* sensu lato exhibits phenotypic plasticity (Barzaghi et al. 2022). Further study is needed to elucidate the specific drivers of this color-pattern polymorphism.

## Supplementary Material

XML Treatment for Hypselotriton (Cynotriton) qianshan

XML Treatment for Hypselotriton (Cynotriton) orientalis

## Additional information

### Artificial Intelligence (AI) use

The authors accept full responsibility for the content of the manuscript, including the disclosure of any use of AI.

No AI tools were used in the preparation of this manuscript.

### Author ORCIDs

Z. Jiang https://orcid.org/0009-0004-1492-0665

W. Zhao https://orcid.org/0009-0009-5702-156X

Y. Pan https://orcid.org/0009-0006-8752-1344

S. Huang https://orcid.org/0000-0001-6786-8523

J. Raffaëlli https://orcid.org/0000-0003-0567-3661

J. Chen https://orcid.org/0000-0001-6432-7721

### Data availability

All of the data that support the findings of this study are available in the main text or Supplementary Information.

## Supplementary materials

Supplementary material 1 Supplementary table Data type xls Explanation note External measurements were made for 47 specimens of *H. orientalis* sensu lato with digital calipers to the nearest 0.1 mm. https://binary.pensoft.net/file/1611234This dataset is made available under the Open Database License (http://opendatacommons.org/licenses/odbl/1.0/). The Open Database License (ODbL) is a license agreement intended to allow users to freely share, modify, and use this Dataset while maintaining this same freedom for others, provided that the original source and author(s) are credited. Zhihao Jiang, Jingjing Yang, Wei Zhao, Beibei He, Yue Pan, Song Huang, Jean Raffaëlli, Jinmin Chen
